# 

**Published:** 1888-12

**Authors:** 


### CONTENTS.

Article I.—A Review of J. C. Reeve, M. D., on "Anæsthetics in Dental Practice." By S. J. Hays ............ 337

" II.—Certain Points Connected With the Structure of Dentine. By F. J. Bennett, M.R.C.S.,L.D.S. 349

" III.—Cocaine in Surgery. By Dr. Freidrich Haenel...... 357

" IV.—Electricity in Dentistry.......................... 363

" V.—Pivot Teeth, Crowns, Etc. By W. St. George Elliott .......................... 366

" VI.—A New Aluminum Process.......................... 373

St. Louis Dental Society.......................... 375

### EDITORIAL.

The University of Maryland Dental Department and the Secretary of the National Association of Dental Examiners.............. 375

Aluminium in the Arts and Manufactures.......................... 381

Hypnotism at Berlin .......................... 382

Biting the Finger Nails .......................... 383

### MONTHLY SUMMARY.

Death by Electricity. The New Method of Executing Criminals. 384

